# Rohingya refugee health and well-being in Malaysia: a call for research and action

**DOI:** 10.1016/j.lanwpc.2024.101229

**Published:** 2024-10-21

**Authors:** Kit-Aun Tan

**Affiliations:** Department of Psychiatry, Faculty of Medicine and Health Sciences, Universiti Putra Malaysia, 43400 UPM Serdang, Selangor Darul Ehsan, Malaysia

Despite the growing numbers and considerable health challenges faced by Rohingya refugees in Malaysia, their health and well-being remain understudied and underfunded. Since the 2017 military crackdown in Myanmar, over 200,000 Rohingya have fled to Malaysia, seeking safety from persecution and human rights violations.^1^ Even though Malaysia is not a signatory to the 1951 Refugee Convention or its 1967 Protocol, it has become a major destination for Rohingya refugees in Southeast Asia.^2^ However, without legal recognition as refugees and with restricted access to formal employment, education, and healthcare, this population faces significant vulnerabilities, highlighting the need for urgent research and collaborative action.^3^

Despite the severity of the humanitarian crisis, research on the health status of Rohingya refugees in Malaysia remains limited—existing studies identify major challenges, particularly concerning communicable diseases and reproductive health. For instance, a study involving refugee children in the Klang Valley, most of whom were born in Myanmar, reported a notable prevalence of latent tuberculosis infection.^4^ In addition, research conducted among Rohingya women attending the IMAM Response and Relief Team Mobile Clinic in Selayang, Selangor, identified significant reproductive health issues, such as pre-eclampsia and gestational diabetes, alongside low contraceptive use and limited knowledge of family planning.^5^ These findings highlight the need for comprehensive epidemiological research to better understand the diverse health needs of Rohingya refugees, taking into account variables such as age and gender, and focusing topics such as communicable and non-communicable diseases, as well as maternal and child health.

Another area of concern that remains inadequately addressed is the mental health of Rohingya refugees in Malaysia. Prolonged exposure to conflict, persecution, and displacement has been found to increase refugees’ vulnerability to mental health issues such as post-traumatic stress disorder, anxiety, depression, and suicidal ideation.^6^ A cross-sectional study of Rohingya refugees in Malaysia revealed that low to moderate social support and food insecurity were strongly associated with major depressive disorder, while exposure to violence and food insecurity were linked to post-traumatic stress disorder.^7^ Future research efforts should aim to investigate the broader social determinants of health that disproportionately affect the mental health of Rohingya refugees, including inadequate living conditions and food insecurity. Interventions focused on improving living conditions, enhancing food security, and delivering culturally and linguistically sensitive psychological support must be made widely accessible.^8^

Access to healthcare remains a significant challenge for Rohingya refugees, primarily due to their undocumented status. A qualitative study with healthcare professionals highlighted how social, cultural, and economic barriers, compounded by legal constraints, limit the ability of refugees and asylum seekers to access comprehensive care.^9^ While high-quality healthcare is available in Malaysia, it is largely unaffordable for refugees, particularly those with chronic conditions that require ongoing treatment.^10^ Given these findings, it is crucial to engage the Rohingya refugee community in the research process itself. Utilizing participatory action research methods—involving community leaders, mental health practitioners, and refugee organizations in the design and implementation of research—can ensure that healthcare services are tailored to community's needs and remain affordable.^11^

Ensuring equitable healthcare for refugees requires a multifaceted approach that addresses both legal and barriers. Policy reforms that recognize the legal status of refugees and ensure access to affordable healthcare are crucial in reducing health inequities.^9^ Collaboration between the Malaysian government, UN agencies, NGOs, and the global community is necessary to deliver comprehensive health interventions. Taken together, these interventions must address both immediate healthcare needs and broader social determinants of health, such as socioeconomic integration and legal protections.^12^

Investing in research to explore the health and well-being of Rohingya refugees in Malaysia is not only an ethical obligation but also a strategic priority for regional health security. Addressing the health challenges of this population requires coordinated actions that integrate multiple dimensions vis-à-vis healthcare, legal reform, community engagement, and international cooperation. As countries in the region confront the complex health implications of displacement and migration, prioritizing refugee health is crucial to advancing accessibility, affordability, and equity in healthcare across Southeast Asia.

## Declaration of interests

No funding was obtained for this study. Kit-Aun Tan is the Secretary of the Malaysian Society of Internet Addiction Prevention, the Secretary of the Malaysian Educational and Psychological Wellbeing Society, and a Working Group Member of the Coalition for Equitable Research in Low-Resource Settings. There are no other competing interests to disclose.
